# Expression and Contribution of NLRP3 Inflammasome During the Follicular Development Induced by PMSG

**DOI:** 10.3389/fcell.2019.00256

**Published:** 2019-10-30

**Authors:** Zhenghong Zhang, Fan Wang, Yan Zhang

**Affiliations:** Provincial Key Laboratory for Developmental Biology and Neurosciences, College of Life Sciences, Fujian Normal University, Fuzhou, China

**Keywords:** NLRP3 inflammasome, pregnant mare serum gonadotropin, follicular development, ovulation, mouse

## Abstract

Follicular development and following ovulation induced by luteinizing hormone (LH) surge are critical for ovarian functions, but the molecular mechanism regulating ovarian ovulation attracts more attention and remains mainly unknown. Recent researches on the nucleotide leukin rich polypeptide 3 (NLRP3) inflammasome shred light on it. Given pregnant mare serum gonadotropin (PMSG) can not only trigger the follicular development, but also induce the following ovulation, the present study therefore examined that expression and localization of NLRP3 inflammasome through immunohistochemistry and Western blotting during the follicular development induced by PMSG. The results showed expressions of NLRP3 and the adaptor protein apoptosis-associated speck-like protein (ASC) significantly increased in the outside of intrafollicular fluid, further analysis found that caspase-1 was activated and IL-1β production was also upregulated after 52 h-treatment of PMSG. Furthermore, a significant increase of ovulation-related genes, hypoxia inducible factor (HIF)-1α and endothelin (ET)-1, was found after 52 h-treatment of PMSG. To our knowledge, it is the first time to clearly indicated the activation of NLRP3 inflammasome may contribute to the ovulation of PMSG-treated ovaries, which will help to further clarify the ovulatory mechanism in mammals.

## Introduction

In mammals, the ovary is the reproductive organs of females, and its main function is to produce oocytes and steroids. At present, it’s believed that follicular development is triggered by follicle-stimulating hormone (FSH) and the following ovulation is induced by luteinizing hormone (LH) surge (Komatsu and Masubuchi, 2016; Jiang et al., 2018; Qi et al., 2018; Wei et al., 2018). Pregnant mare serum gonadotropin (PMSG) can not only trigger the follicular development mainly similar to FSH, but also induce the following ovulation like LH, which was widely used in the field of reproductive investigation (Ma et al., 1997; Tarín et al., 2002; Nie et al., 2018; Kim et al., 2019). It is worth noting that Espey put forward the hypothesis of ovulation as an inflammatory reaction (Espey, 1980), but the detailed mechanism regulating ovarian ovulation still remains unknown. Until recently, the researches on the nucleotide leukin rich polypeptide 3 (NLRP3) inflammasome shred light on it (Ahechu et al., 2018; Groslambert and Py, 2018; de Alba, 2019; Komada and Muruve, 2019; Takahashi, 2019; Yang et al., 2019).

The inflammasome is the cellular machinery responsible for activation of inflammatory reaction (Mariathasan et al., 2004; Zhang et al., 2012; Li et al., 2014), which includes four types, like NLRP1, NLRP3, IPAF, and AIM2 inflammasome (Boini et al., 2014; Abais et al., 2015; Chen et al., 2016). NLRP3 inflammasome is a proteolytic complex of NLRP3, ASC, and caspase-1, which is well characterized (Boini et al., 2014; Abais et al., 2015; Chen et al., 2015; Zhang et al., 2015; Chen et al., 2016; Wang et al., 2016). Upon activation, NLRP3 binds to the ASC, which in turn recruits pro-caspase-1 to form an integrated inflammasome complex (Zhang et al., 2012; Boini et al., 2014; Li et al., 2014; Abais et al., 2015; Chen et al., 2015, 2016; Zhang et al., 2015; Wang et al., 2016), and subsequently cleaves pro-caspase-1 into activated caspase-1, leading to the production of matured IL-1β (Zhang et al., 2012; Boini et al., 2014; Li et al., 2014; Abais et al., 2015; Chen et al., 2015; Zhang et al., 2015; Chen et al., 2016; Wang et al., 2016), which is an important inflammatory cytokine (Zhang et al., 2012; Boini et al., 2014; Li et al., 2014; Abais et al., 2015; Chen et al., 2015; Zhang et al., 2015; Chen et al., 2016; Wang et al., 2016). However, the expression and contribution of NLRP3 Inflammasome during follicular development and ovarian ovulation still unknown.

Given the parallels of ovulation with inflammatory processes and the correlation of non-steroidal anti-inflammatory drug use with reversible infertility (Akil et al., 1996; Mendonça et al., 2000; Duffy et al., 2019), the present study therefore utilized animal model to examine that expression and localization of NLRP3 inflammasome through immunohistochemistry and Western blotting during the follicular development induced by PMSG for help to further clarify the ovulatory mechanism in mammals.

## Materials and Methods

### Experimental Design

Immature female C57BL/6 mice (21-day old) were purchased from Wushi Experimental Animal Supply Co. Ltd. (Fuzhou, China) and maintained in the Laboratory Animal Center of Fujian Normal University under a 14-h light/10-h dark schedule with continuous supply of chow and water. The follicular development was induced in the mice treated with 10 IU PMSG (i.p., Sigma-Aldrich, St. Louis, MO, United States) for 0, 24, and 52 h. The left ovary of each animal was fixed in 4% paraformaldehyde for immunohistochemistry, and the right one was snap-frozen for the examination of gene and protein expression levels. The experiment was repeated two times.

### Immunohistochemistry

After fixation, 5-μm sections were processed for immunohistochemical analysis with anti-NLRP3 antibody (1:500, Abcam, Cambridge, MA, United States), anti-ASC antibody (1:500, Abcam, Cambridge, MA, United States) and anti-IL-1β antibody (1:500, Abcam, Cambridge, MA, United States). The negative control used serum (Boster Biological Technology, Wuhan, China) instead of primary antibody and the immunoreactivity was visualized by the Elite ABC kit (BioGenex, San Ramon, CA, United States). Three independent observers were asked to assess the intensity of immunostaining (Shi et al., 2004; Kim et al., 2005) and the evaluation of relative staining levels was repeated at least four times (Zhang W. et al., 2011).

### Western Blot Analysis

Cytoplasmic protein extracts were prepared with the Protein Extraction Kit (Beyotime Biotechnology, Shanghai, China) according to the manufacturer’s instructions, and the concentrations were determined by a BCA assay kit (Beyotime Biotechnology, Shanghai, China). These proteins were used for the following examination of expression levels and activities. The expression levels of different proteins were analyzed by Western blotting as described previously (Zhang Z. et al., 2011). NLRP3 antibody (1:1000, Abcam, Cambridge, MA, United States), ASC antibody 1:1000, Abcam, Cambridge, MA, United States), Pro-caspase-1 antibody (1:1000, Abcam, Cambridge, MA, United States), cleaved-caspase-1 antibody (1:1000, Abcam, Cambridge, MA, United States), IL-1β antibody (1:500, Abcam, Cambridge, MA, United States) and β-actin antibody (1:5000, Novus Biologicals, Littleton, CO, United States) were used.

### Real Time PCR Analysis

Total RNA was extracted using TRIzol (Life Technologies, Carlsbad, CA, United States) and then reverse-transcribed (cDNA Synthesis Kit, Bio-Rad, Hercules, CA, United States). The reverse-transcribed products were amplified using a TaqMan Gene Expression Assay kit (Applied Biosystems, Foster City, CA, United States), including TaqMan Universal PCR Master Mix, hypoxia-inducible factor (HIF)-1α primer (Hs00936372_m1) and endothelin (ET)-1 primer (Mm00438656_m1). The levels of 18S ribosomal RNA (Rn03928990_g1) was used as an endogenous control. The relative gene expressions were calculated with the ΔΔCt method. Relative mRNA levels were expressed as 2^–ΔΔCt^ values.

### Caspase-1 Activity and IL-1β Production Assay

The assay of caspase-1 activity was performed by Caspase-1 Colorimetric Assay Kit (Biovision, Milpitas, CA, United States) and the level of IL-1β production was measured by IL-1β ELISA Kit (Bender Medsystems, Burlingame, CA, United States) according to the protocol described by the manufacturer. These data were expressed as the fold change compared with the control.

### Statistics

Data are presented as mean ± SE. The significance of differences in mean values within multiple groups was evaluated using a one-way analysis of variance (ANOVA), followed by a Tukey’s multiple range test. *P* < 0.05 was considered statistically significant.

## Results

### Immunohistochemical Analysis of NLRP3 Inflammasomes

**FIGURE 1:**
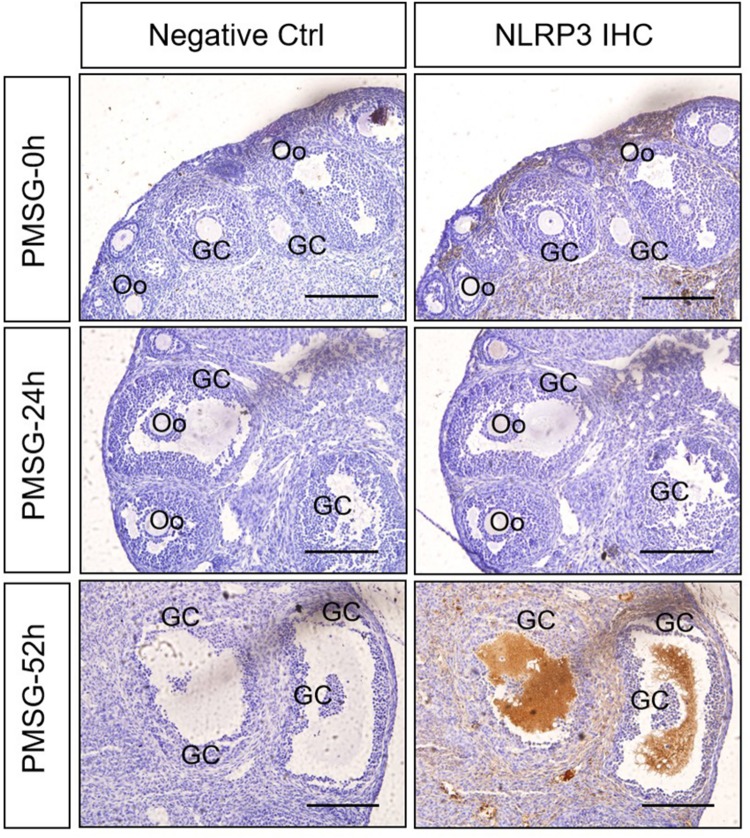
NLRP3 immunohistochemistry in the ovary during the follicular development induced by PMSG Ovarian sections were immunostained for NLRP3 and counterstained with hematoxylin. The NLRP3 immunohistochemical signals appear brown, and the background counterstaining appears blue. Negative control remained unstained, lacking primary antibody instead of serum. GC, granulosa cell; Oo, oocyte; bar = 100 μm.

**FIGURE 2:**
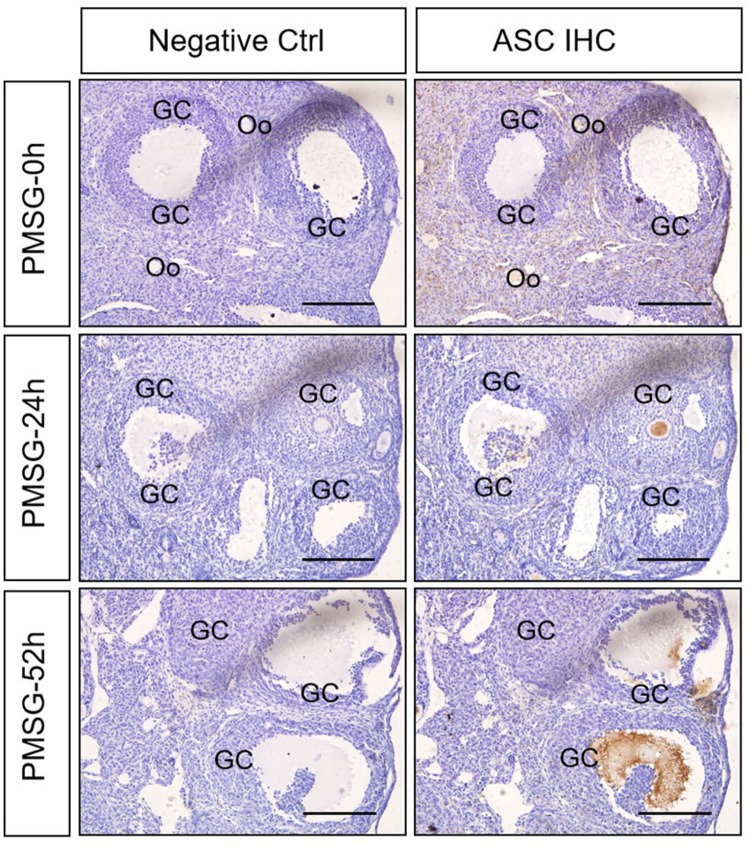
ASC immunohistochemistry in the ovary during the follicular development induced by PMSG Ovarian sections were immunostained for ASC and counterstained with hematoxylin. The ASC immunohistochemical signals appear brown, and the background counterstaining appears blue. Negative control remained unstained, lacking primary antibody instead of serum. GC, granulosa cell; Oo, oocyte; bar = 100 μm.

In the present study, the localization of the core protein NLRP3 and the adaptor protein ASC of inflammasomes were examined through immunohistochemical staining (Figures 1, 2), and the relative expressions were present in Tables 1, 2. The results showed NLRP3 mainly expressed in the outside of intrafollicular fluid in the ovaries with PMSG-52 h treatment (Figure 1), which was similar with the pattern of ASC expressions (Figure 2).

**TABLE 1:** Relative abundances of NLRP3 in the ovary during follicular development induced by PMSG.

	**Staining intensity**		
**Follicular development**	**PMSG-0 h**	**PMSG-24 h**	**PMSG-52 h**
Oocyte			
Primordial	−	NA	NA
Primary	−	−	+
Secondary	−	+	+
Tertiary	NA	NA	+
Granulosa cells			
Primordial	−	NA	NA
Primary	−	−	−
Secondary	−	−	−
Tertiary	NA	NA	−
Theca cells			
Secondary	+	+	+
Tertiary	NA	NA	+
Endothelial cells	+	+	++
Follicular fluid	−	−	+++

*−, no staining detected; +, weak; ++, moderate; +++, strong; NA, not available.*

**TABLE 2:** Relative abundances of ASC in the ovary during follicular development induced by PMSG.

**Follicular development**	**Staining intensity**		
	**PMSG-0 h**	**PMSG-24 h**	**PMSG-52 h**
Oocyte			
Primordial	−	NA	NA
Primary	−	−	+
Secondary	+	++	++
Tertiary	NA	NA	++
Granulosa cells			
Primordial	−	NA	NA
Primary	−	−	−
Secondary	−	−	−
Tertiary	NA	NA	−
Theca cells			
Secondary	−	−	−
Tertiary	NA	NA	−
Endothelial cells	−	−	−
Follicular fluid	−	−	+++

*−, no staining detected; +, weak; ++, moderate; +++, strong; NA, not available.*

### Expression Changes of NLRP3 Inflammasomes in the Ovary During the Follicular Development Induced by PMSG

**FIGURE 3:**
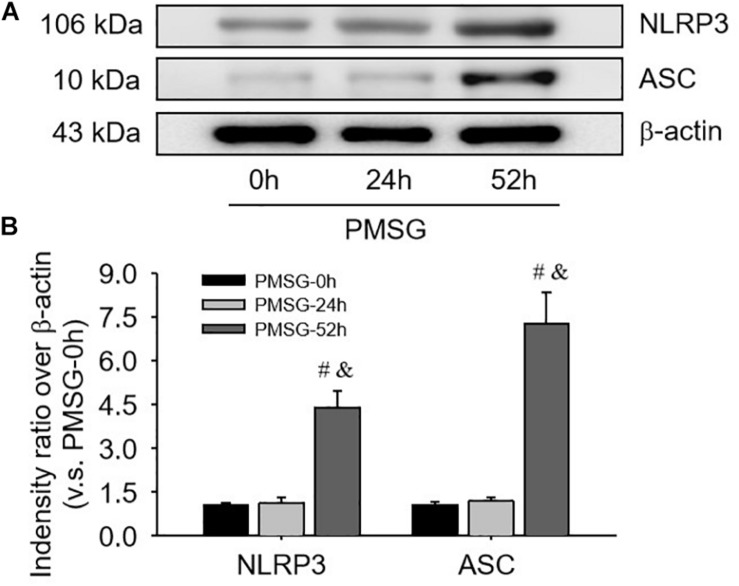
NLRP3 and ASC protein expressions in the ovary during the follicular development induced by PMSG. **(A)** Representative ECL gel images of Western blot analyses depicting the NLRP3 and ASC protein levels. **(B)** Summarized intensities of NLRP3 and ASC blots normalized to the control. Each value represents the mean ± SE. One-way analysis of variance (ANOVA) was used to analyze the data, followed by a Tukey’s multiple range test. *n* = 6. ^#^*P* < 0.05, vs. PMSH-0 h; ^&^*P* < 0.05, vs. PMSH-24 h.

**FIGURE 4:**
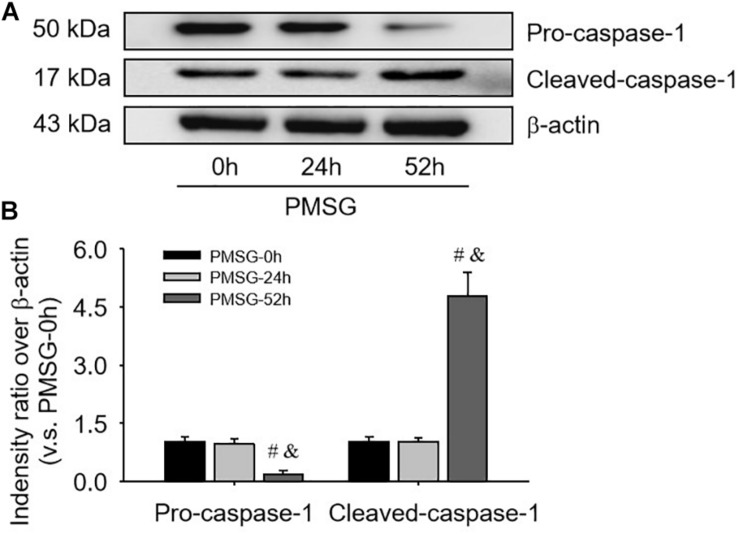
Pro-caspase-1 and cleaved-caspase-1 protein expressions in the ovary during the follicular development induced by PMSG. **(A)** Representative ECL gel images of Western blot analyses depicting the pro-caspase-1 and cleaved-caspase-1 protein levels. **(B)** Summarized intensities of pro-caspase-1 and cleaved-caspase-1 blots normalized to the control. Each value represents the mean ± SE. One-way analysis of variance (ANOVA) was used to analyze the data, followed by a Tukey’s multiple range test. *n* = 6. ^#^*P* < 0.05, vs. PMSH-0 h; ^&^*P* < 0.05, vs. PMSH-24 h.

For confirming the above findings, the expressions of NLRP3 and ASC were further examined by Western blotting (Figure 3) and the results also suggested NLRP3 and ASC mainly expressed in the ovaries after PMSG-52 h treatment, indicating the activation of NLRP3 inflammasomes this time. Therefore, the expressions of cleaved-caspase-1 were detected (Figure 4) and then found a significant decrease of pro-caspase-1 expression and a dramatic increase of cleaved-caspase-1 expression in the ovaries after PMSG-52 h treatment (Figure 4), implying NLRP3 inflammasomes may be involved in the following ovulation induced by MPSG.

### Expression and Localization of IL-1β in the Ovary During the Follicular Development Induced by PMSG

**FIGURE 5:**
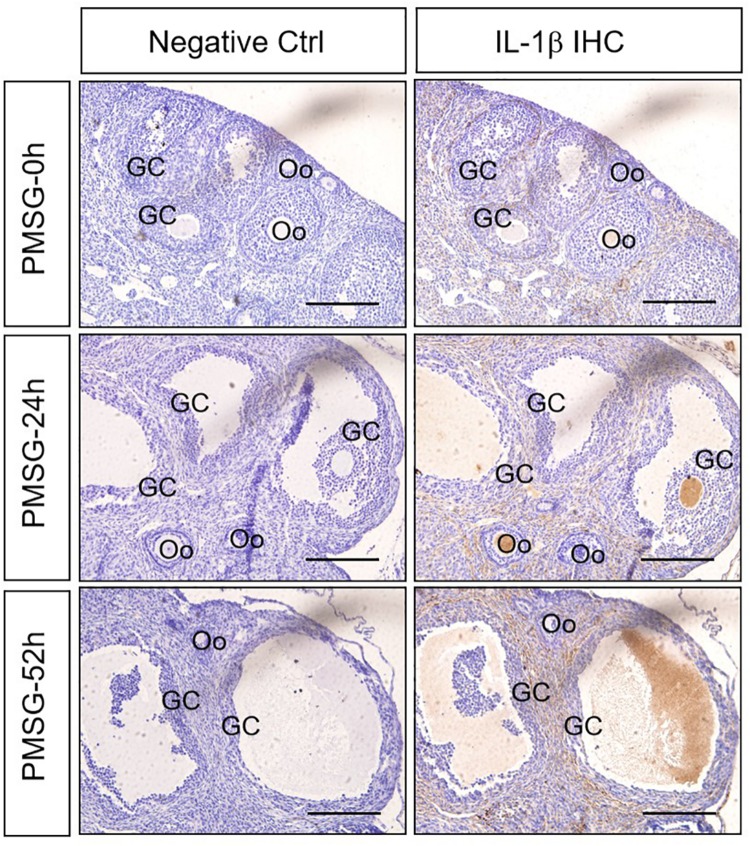
IL-1β Immunohistochemistry in the ovary during the follicular development induced by PMSG Ovarian sections were immunostained for IL-1β and counterstained with hematoxylin. The IL-1β immunohistochemical signals appear brown, and the background counterstaining appears blue. Negative control remained unstained, lacking primary antibody instead of serum. GC, granulosa cell; Oo, oocyte; bar = 100 μm.

**FIGURE 6:**
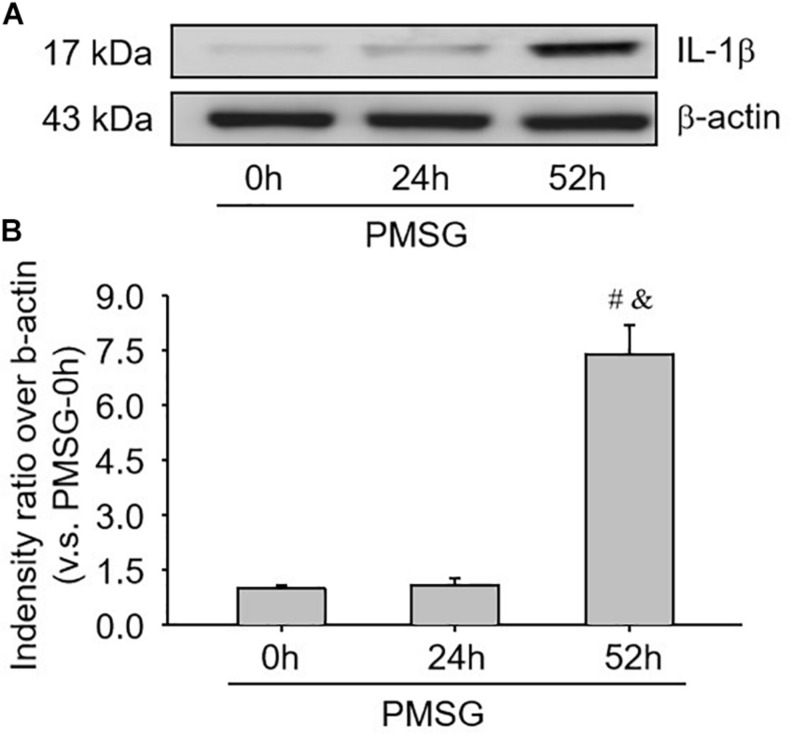
IL-1β protein expressions in the ovary during the follicular development induced by PMSG. **(A)** Representative ECL gel images of Western blot analyses depicting the IL-1β protein levels. **(B)** Summarized intensities of IL-1β blots normalized to the control. Each value represents the mean ± SE. One-way analysis of variance (ANOVA) was used to analyze the data, followed by a Tukey’s multiple range test. *n* = 6. ^#^*P* < 0.05, vs. PMSH-0 h; ^&^*P* < 0.05, vs. PMSH-24 h.

Given IL-1β production resulted from the activation of NLRP3 inflammasomes, the present study examined the expression (Figure 5 and Table 3) and localization (Figure 5) of IL-1β in the ovary during the follicular development induced by PMSG and the results further demonstrated IL-1β mainly expressed in the outside of intrafollicular fluid (Figure 5) and significantly increased (Figure 6) in the ovaries with PMSG-52 h treatment, which were similar with the expression pattern of NLRP3 and ASC proteins.

**TABLE 3:** Relative abundances of IL-1β in the ovary during follicular development induced by PMSG.

**Follicular development**	**Staining intensity**		
	**PMSG-0 h**	**PMSG-24 h**	**PMSG-52 h**
Oocyte			
Primordial	−	NA	NA
Primary	+	+	+
Secondary	++	+++	+++
Tertiary	NA	NA	+++
Granulosa cells			
Primordial	−	NA	NA
Primary	−	−	−
Secondary	−	−	−
Tertiary	NA	NA	−
Theca cells			
Secondary	+	+	+
Tertiary	NA	NA	++
Endothelial cells	−	−	−
Follicular fluid	−	−	+++

*−, no staining detected; +, weak; ++, moderate; +++, strong; NA, not available.*

### Activity Changes of Caspase-1 in the Ovary During the Follicular Development Induced by PMSG

**FIGURE 7:**
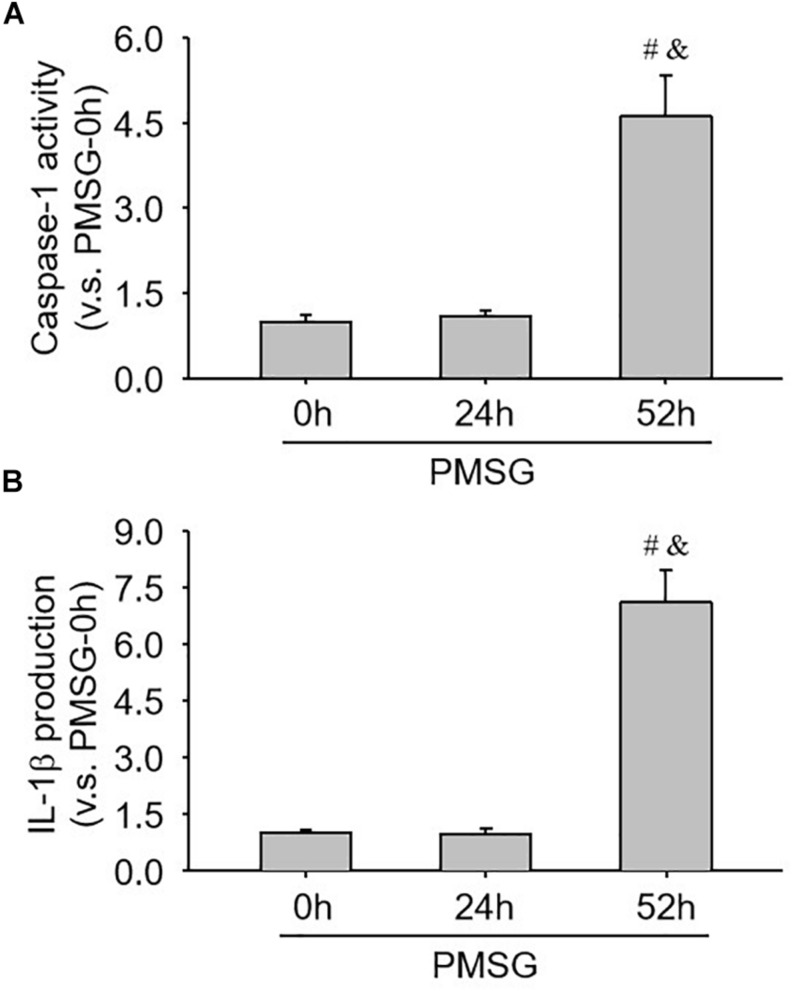
Examination of caspase-1 activity and IL-1β production in the ovary during the follicular development induced by PMSG. **(A)** The data for caspase-1 activity in the ovaries from each group normalized to the control (PMSG-0 h). **(B)** The data for IL-1β production in the ovaries from each group normalized to the control (PMSG-0 h). Each value represents the mean ± SE. One-way analysis of variance (ANOVA) was used to analyze the data, followed by a Tukey’s multiple range test. *n* = 6. ^#^*P* < 0.05, vs. PMSH-0 h; ^&^*P* < 0.05, vs. PMSH-24 h.

Furthermore, the present study also examined caspase-1 activity (Figure 7A) and IL-1b production (Figure 7B) through ELISA kits and further found a significant increase of caspase-1 activity (Figure 7A) and a dramatic increase of IL-1β production (Figure 7B), suggesting NLRP3 inflammasomes were activated and involved in the following ovulation induced by MPSG.

### Expression Changes of Ovulation-Related Genes in the Ovary During the Follicular Development Induced by PMSG

**FIGURE 8:**
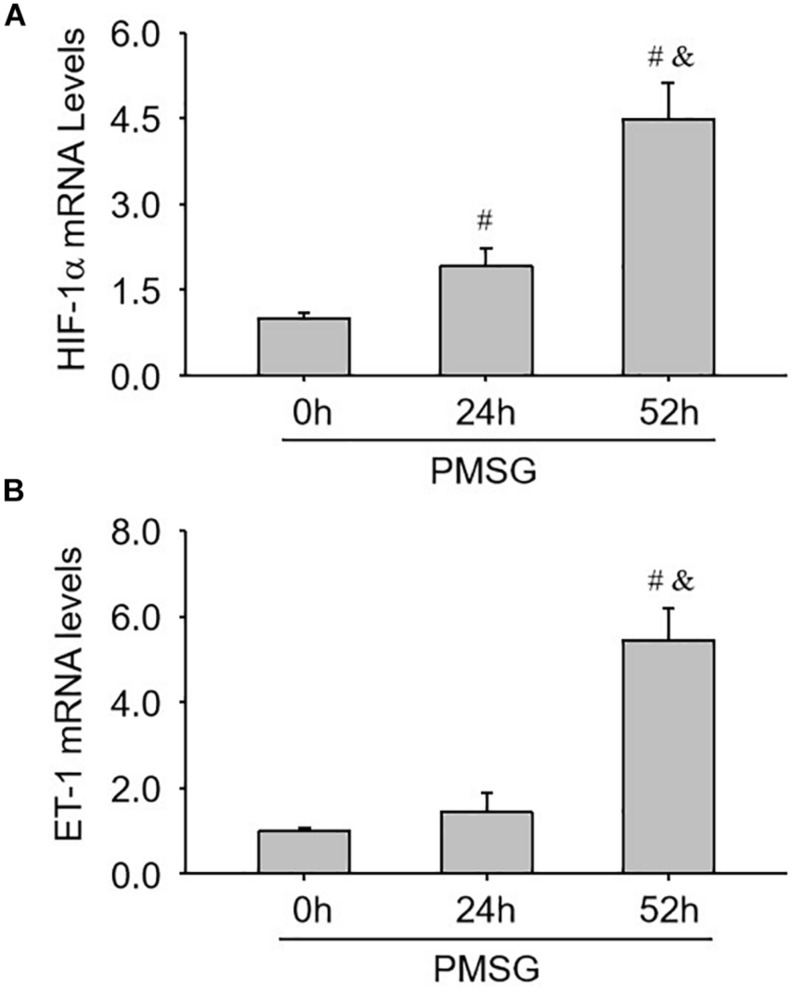
Expression changes of HIF-1α and ET-1 mRNA in the ovaries during the follicular development induced by PMSG. **(A)** The relative mRNA levels of HIF-1α by real-time PCR analysis. **(B)** The relative mRNA levels of ET-1 by real-time PCR analysis. Each value represents the mean ± SE. One-way analysis of variance (ANOVA) was used to analyze the data, followed by a Tukey’s multiple range test. *n* = 6. ^#^*P* < 0.05, vs. PMSH-0 h; ^&^*P* < 0.05, vs. PMSH-24 h.

**FIGURE 9:**
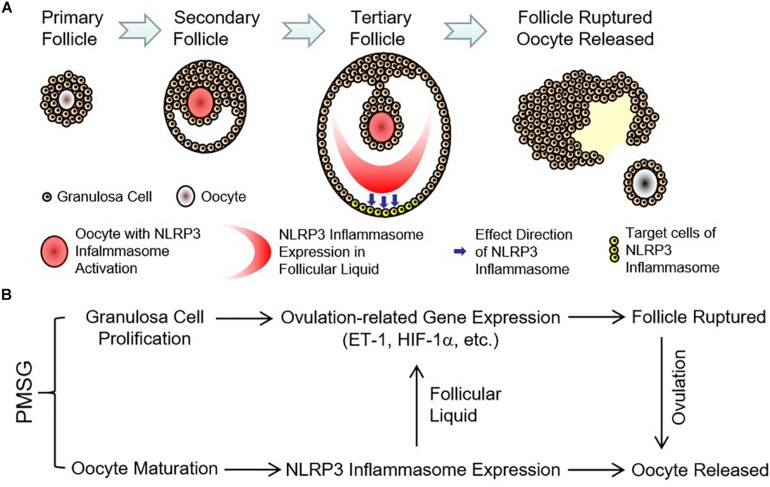
Expression pattern and possible role of NLRP3 inflammasomes in the ovaries during the follicular development induced by PMSG. **(A)** Expression pattern of NLRP3 inflammasomes in the ovarian follicles during the follicular development induced by PMSG. **(B)** Possible role of NLRP3 inflammasomes in the PMSG-treated ovaries during the follicular development and the following ovulation.

Finally, the expressions of ovulation-related genes, HIF-1α (Figure 8A) and ET-1 (Figure 8B), were examined through real time PCR and found their expressions also increased significantly in the ovaries with PMSG-52 h treatment (Figure 8), suggesting the activation of NLRP3 inflammasomes may take participate in the ovulatory process with the detailed mechanism to be further clarified. Together, the present study not only summarized the expression changes of NLRP3 inflammasomes during the follicular development (Figure 9A), but also put forward the possible role of NLRP3 inflammasomes during the following ovulation (Figure 9B).

## Discussion

The present study mainly examined that expression of NLRP3 inflammasomes during the follicular development induced by PMSG, clearly demonstrating that the activation of NLRP3 inflammasomes may take participate in the following process of ovulation, which will help to further clarify the ovulatory mechanism in mammals.

Pregnant mare serum gonadotropin is mainly similar to FSH and widely used for superovulation (Ma et al., 1997; Tarín et al., 2002; Nie et al., 2018; Kim et al., 2019), the present study therefore, utilized PMSG to trigger the follicular development and the following ovulation for examining the expression changed of NLRP3 inflammasomes during the follicular development and clarifying the possible role of NLRP3 inflammasomes during the following ovulation. It’s well-known that inflammation is involved in the ovulatory process (Gonzalez et al., 2018; Duffy et al., 2019; Poulsen et al., 2019; Snider and Wood, 2019), but the detailed mechanism regulating the crosstalk between the inflammatory and ovulatory processes still remains unclear. Therefore, the contribution of the inflammatory processes to the ovulation in mammalian ovaries recently attracted more and more attention (Gonzalez et al., 2018; Duffy et al., 2019; Poulsen et al., 2019; Snider and Wood, 2019). Duffy et al. (2019) Found the parallel of the ovulatory and inflammatory processes with many common features, ovarian granulosa cells executed an inflammatory reaction during the ovulation (Poulsen et al., 2019), obesity reduced ovarian oocyte quality with inflammation (Snider and Wood, 2019), inflammatory markers in the follicular fluid were correlated with body mass index (Gonzalez et al., 2018), and so on. Given the important role of NLRP3 inflammasomes in the inflammatory responses (Zhang et al., 2015; Zhu et al., 2016), the present study examined expressions of NLRP3 inflammasomes during the follicular development induced by PMSG and found NLRP3 and ASC expressions significantly increased after PMSG-52 h treatment, which were consistent with the dramatic increases of caspase-1 activity and IL-1β production, suggesting NLRP3 inflammasomes were activated before the ovulation and involved in the following ovulation. This findings of NLRP3 inflammasome activation could be a new mechanism regulating the ovulatory process in mammals.

Notably, the present study also found that expression and activation of NLRP3 inflammasomes in ovarian oocytes were prior to those in the follicular fluid, suggesting which was regulated by oocyte quality or maturation. Recent investigations have also demonstrated the coordination of ovulation with oocyte maturation and the decisive effects of oocytes during the ovulatory process (Robker et al., 2018). Interestingly, the present results showed that expressions and activation of NLRP3 inflammasomes in the outside of intrafollicular fluid after PMSG-52 h treatment, suggesting the regional responses in the ovulatory follicles (Duffy et al., 2019), which contributed to the apical stretching and thinning (Duffy et al., 2019). Previous studies have indicated ETs facilitated follicular rupture at the apex (Ko et al., 2006; Choi et al., 2011; Cacioppo et al., 2017) and HIF-1α regulated ovulation-related gene expression (Zhang Z. et al., 2011; Wang et al., 2012; Wang et al., 2015) during the ovulatory process. Therefore, the present study further examined the expressions of HIF-1α and ET-1 and found their expression patterns were similar with NLRP inflammasomes, demonstrating the possible contribution of NLRP inflammasomes to the ovulation.

Furthermore, the correlation between non-steroidal anti-inflammatory drug use and reversible infertility in women indicated the ovulatory processes with inflammatory responses (Akil et al., 1996; Mendonça et al., 2000; Duffy et al., 2019), further implying the activation of NLRP3 inflammasomes may play an important role in the regulation of ovarian ovulation in mammals. Although the present findings is very interesting and helpful for further understanding the molecular regulation of the ovulatory process, some more detailed investigations need to be performed in the near future for finally clarifying the relationship between the activation of NLRP3 inflammasomes and the ovulation of mammalian ovaries.

In summarize, the present study firstly demonstrated the expression and activation of NLRP3 inflammasomes during the follicular development induced by PMSG, which may contribute to the following ovulation. Furthermore, enhanced understanding of the contribution and regulation of NLRP3 inflammasomes during ovarian ovulation will be helpful to treat anovulatory infertility, like luteinized unruptured follicle syndrome and polycystic ovary syndrome.

## Data Availability Statement

The datasets generated for this study are available on request to the corresponding author.

## Ethics Statement

The experimental protocol was approved in accordance with the Guide for the Care and Use of Laboratory Animals prepared by the Institutional Animal Care and Use Committee, Fujian Normal University (IACUC-20180011).

## Author Contributions

ZZ and FW designed the study. FW and YZ wrote the manuscript. ZZ revised the manuscript. All authors performed the experiments, analyzed the data, interpreted the data, discussed the results, read, and approved the final version of the manuscript for publication.

## Conflict of Interest

The authors declare that the research was conducted in the absence of any commercial or financial relationships that could be construed as a potential conflict of interest.

## References

[B1] Abais J. M. Xia M. Zhang Y. Boini K. M. Li P. L. (2015). Redox regulation of NLRP3 inflammasomes: ROS as trigger or effector? *Antioxid. Redox. Signal.* 22 1111–1129. 10.1089/ars.2014.5994 25330206 PMC4403231

[B2] Ahechu P. Zozaya G. Martí P. Hernández-Lizoáin J. L. Baixauli J. Unamuno X. (2018). NLRP3 Inflammasome: a possible link between obesity-associated low-grade chronic inflammation and colorectal cancer development. *Front. Immunol.* 9:2918. 10.3389/fimmu.2018.02918 30619282 PMC6297839

[B3] Akil M. Amos R. S. Stewart P. (1996). Infertility may sometimes be associated with NSAID consumption. *Br. J. Rheumatol.* 35 76–78. 8624628 10.1093/rheumatology/35.1.76

[B4] Boini K. M. Xia M. Abais J. M. Li G. Pitzer A. L. Gehr T. W. (2014). Activation of inflammasomes in podocyte injury of mice on the high fat diet: effects of ASC gene deletion and silencing. *Biochim. Biophys. Acta.* 1843 836–845. 10.1016/j.bbamcr.2014.01.033 24508291 PMC3986924

[B5] Cacioppo J. A. Lin P. P. Hannon P. R. McDougle D. R. Gal A. Ko C. (2017). Granulosa cell endothelin-2 expression is fundamental for ovulatory follicle rupture. *Sci. Rep.* 7:817. 10.1038/s41598-017-00943-w 28400616 PMC5429765

[B6] Chen Y. Pitzer A. L. Li X. Li P. L. Wang L. Zhang Y. (2015). Instigation of endothelial Nlrp3 inflammasome by adipokine visfatin promotes inter-endothelial junction disruption: role of HMGB1. *J. Cell. Mol. Med.* 19 2715–2727. 10.1111/jcmm.12657 26293846 PMC4687695

[B7] Chen Y. Wang L. Pitzer A. L. Li X. Li P. L. Zhang Y. (2016). Contribution of redox-dependent activation of endothelial Nlrp3 inflammasomes to hyperglycemia-induced endothelial dysfunction. *J. Mol. Med.* 94 1335–1347. 10.1007/s00109-016-1481-5 27783111 PMC5512566

[B8] Choi D. H. Kim E. K. Kim K. H. Lee K. A. Kang D. W. Kim H. Y. (2011). Expression pattern of endothelin system components and localization of smooth muscle cells in the human pre-ovulatory follicle. *Hum. Reprod.* 26 1171–1180. 10.1093/humrep/der066 21406445 PMC3079471

[B9] de Alba E. (2019). Structure, interactions and self-assembly of ASC-dependent inflammasomes. *Arch. Biochem. Biophys.* 670 15–31. 10.1016/j.abb.2019.05.023 31152698 PMC8455077

[B10] Duffy D. M. Ko C. Jo M. Brannstrom M. Curry T. E. (2019). Ovulation: parallels with inflammatory processes. *Endocr. Rev.* 40 369–416. 10.1210/er.2018-2075 30496379 PMC6405411

[B11] Espey L. L. (1980). Ovulation as an inflammatory reaction–a hypothesis. *Biol. Reprod.* 22 73–106. 10.1095/biolreprod22.1.73 6991013

[B12] Gonzalez M. B. Lane M. Knight E. J. Robker R. L. (2018). Inflammatory markers in human follicular fluid correlate with lipid levels and body mass index. *J. Reprod. Immunol.* 130 25–29. 10.1016/j.jri.2018.08.005 30174020

[B13] Groslambert M. Py B. F. (2018). Spotlight on the NLRP3 inflammasome pathway. *J. Inflamm. Res.* 11 359–374. 10.2147/JIR.S141220 30288079 PMC6161739

[B14] Jiang J. Qi L. Wei Q. Shi F. (2018). Effects of daily exposure to saccharin sodium and rebaudioside a on the ovarian cycle and steroidogenesis in rats. *Reprod. Toxicol.* 76 35–45. 10.1016/j.reprotox.2017.12.006 29262312

[B15] Kim H. Moon C. Ahn M. Lee Y. Kim H. Kim S. (2005). Expression of nitric oxide synthase isoforms in the porcine ovary during follicular development. *J. Vet. Sci.* 6 97–101. 15933428

[B16] Kim J. Sun S. Lee D. Youk H. Yang H. (2019). Gonadotropin regulates NUCB2/nesfatin-1 expression in the mouse ovary and uterus. *Biochem. Biophys. Res. Commun.* 513 602–607. 10.1016/j.bbrc.2019.04.008 30981497

[B17] Ko C. Gieske M. C. Al-Alem L. Hahn Y. Su W. Gong M. C. (2006). Endothelin-2 in ovarian follicle rupture. *Endocrinology* 147 1770–1779. 10.1210/en.2005-1228 16410304

[B18] Komada T. Muruve D. A. (2019). The role of inflammasomes in kidney disease. *Nat. Rev. Nephrol.* 15 501–520. 10.1038/s41581-019-0158-z 31164720

[B19] Komatsu K. Masubuchi S. (2016). Observation of the dynamics of follicular development in the ovary. *Reprod. Med. Biol.* 16 21–27. 10.1002/rmb2.12010 29259446 PMC5715870

[B20] Li X. Zhang Y. Xia M. Gulbins E. Boini K. M. Li P. L. (2014). Activation of Nlrp3 inflammasomes enhances macrophage lipid-deposition and migration: implication of a novel role of inflammasome in atherogenesis. *PLoS One* 9:e87552. 10.1371/journal.pone.0087552 24475307 PMC3903678

[B21] Ma S. Kalousek D. K. Yuen B. H. Moon Y. S. (1997). Investigation of effects of pregnant mare serum gonadotropin (PMSG) on the chromosomal complement of CD-1 mouse embryos. *J. Assist. Reprod. Genet.* 14 162–169. 10.1007/bf02766134 9090560 PMC3454672

[B22] Mariathasan S. Newton K. Monack D. M. Vucic D. French D. M. Lee W. P. (2004). Differential activation of the inflammasome by caspase-1 adaptors ASC and Ipaf. *Nature* 430 213–218. 10.1038/nature02664 15190255

[B23] Mendonça L. L. Khamashta M. A. Nelson-Piercy C. Hunt B. J. Hughes G. R. (2000). Non-steroidal anti-inflammatory drugs as a possible cause for reversible infertility. *Rheumatology* 39 880–882. 10.1093/rheumatology/39.8.880 10952743

[B24] Nie X. Dai Y. Zheng Y. Bao D. Chen Q. Yin Y. (2018). Establishment of a mouse model of premature ovarian failure using consecutive superovulation. *Cell. Physiol. Biochem.* 51 2341–2358. 10.1159/000495895 30537739

[B25] Poulsen L. C. Englund A. L. M. Wissing M. L. M. Yding Andersen C. Borup R. Grøndahl M. L. (2019). Human granulosa cells function as innate immune cells executing an inflammatory reaction during ovulation: a microarray analysis. *Mol. Cell. Endocrinol.* 486 34–46. 10.1016/j.mce.2019.02.014 30802528

[B26] Qi L. Jiang J. Jin P. Kuang M. Wei Q. Shi F. (2018). Expression patterns of claudin-5 and its related signals during luteal regression in pseudopregnant rats: the enhanced effect of additional PGF treatment. *Acta. Histochem.* 120 221–227. 10.1016/j.acthis.2018.02.001 29449022

[B27] Robker R. L. Hennebold J. D. Russellm D. L. (2018). Coordination of ovulation and oocyte maturation: a good egg at the right time. *Endocrinology* 159 3209–3218. 10.1210/en.2018-2485 30010832 PMC6456964

[B28] Shi F. Stewart R. L. Jr. Perez E. Chen J. Y. LaPolt P. S. (2004). Cell-specific expression and regulation of soluble guanylyl cyclase alpha 1 and beta 1 subunits in the rat ovary. *Biol. Reprod.* 70 1552–1561. 10.1095/biolreprod.103.025510 14749300

[B29] Snider A. P. Wood J. R. (2019). Obesity induces ovarian inflammation and reduces oocyte quality. *Reproduction* 10.1530/REP-18-0583 [Epub ahead of print]. 30999278

[B30] Takahashi M. (2019). Role of NLRP3 inflammasome in cardiac inflammation and remodeling after myocardial infarction. *Biol. Pharm. Bull.* 42 518–523. 10.1248/bpb.b18-00369 30930410

[B31] Tarín J. J. Pérez-Albalá S. Cano A. (2002). Stage of the estrous cycle at the time of pregnant mare’s serum gonadotropin injection affects the quality of ovulated oocytes in the mouse. *Mol. Reprod. Dev.* 61 398–405. 10.1002/mrd.10042 11835585

[B32] Wang F. Zhang Z. Wang Z. Xiao K. Wang Q. Su J. (2015). Expression and clinical significance of the HIF-1α/ET-2 signaling pathway during the development and treatment of polycystic ovary syndrome. *J. Mol. Histol.* 46 173–181. 10.1007/s10735-015-9609-9604 25613530

[B33] Wang L. Chen Y. Li X. Zhang Y. Gulbins E. Zhang Y. (2016). Enhancement of endothelial permeability by free fatty acid through lysosomal cathepsin B-mediated Nlrp3 inflammasome activation. *Oncotarget* 7 73229–73241. 10.18632/oncotarget.12302 27689324 PMC5341975

[B34] Wang Z. Zhang Z. Wu Y. Chen L. Luo Q. Zhang J. (2012). Effects of echinomycin on endothelin-2 expression and ovulation in immature rats primed with gonadotropins. *Exp. Mol. Med.* 44 615–621. 10.3858/emm.2012.44.10.070 22874467 PMC3490083

[B35] Wei Q. Fedail J. S. Kong L. Zheng K. Meng C. Fadlalla M. B. (2018). Thyroid hormones alter estrous cyclicity and antioxidative status in the ovaries of rats. *Anim. Sci. J.* 89 513–526. 10.1111/asj.12950 29214681

[B36] Yang Q. Liu R. Yu Q. Bi Y. Liu G. (2019). Metabolic regulation of inflammasomes in inflammation. *Immunology* 157 95–109. 10.1111/imm.13056 30851192 PMC6526672

[B37] Zhang C. Boini K. M. Xia M. Abais J. M. Li X. Liu Q. (2012). Activation of nod-like receptor protein 3 inflammasomes turns on podocyte injury and glomerular sclerosis in hyperhomocysteinemia. *Hypertension* 60 154–162. 10.1161/HYPERTENSIONAHA.111.189688 22647887 PMC3753400

[B38] Zhang W. Wei Q. W. Wang Z. C. Ding W. Wang W. Shi F. X. (2011). Cell-specific expression and immunolocalization of nitric oxide synthase isoforms and the related nitric oxide/cyclic GMP signaling pathway in the ovaries of neonatal and immature rats. *J. Zhejiang Univ. Sci. B* 12 55–64. 10.1631/jzus.B1000174 21194187 PMC3017417

[B39] Zhang Y. Li X. Pitzer A. L. Chen Y. Wang L. Li P. L. (2015). Coronary endothelial dysfunction induced by nucleotide oligomerization domain-like receptor protein with pyrin domain containing 3 inflammasome activation during hypercholesterolemia: beyond inflammation. *Antioxid. Redox. Signal.* 22 1084–1096. 10.1089/ars.2014.5978 25739025 PMC4403230

[B40] Zhang Z. Yin D. Wang Z. (2011). Contribution of hypoxia-inducible factor-1a to transcriptional regulation of vascular endothelial growth factor in bovine developing luteal cells. *Anim. Sci. J.* 82 244–250. 10.1111/j.1740-0929.2010.00832.x 21729202

[B41] Zhu Q. Li X. X. Wang W. Hu J. Li P. L. Conley S. (2016). Mesenchymal stem cell transplantation inhibited high salt-induced activation of the NLRP3 inflammasome in the renal medulla in Dahl S rats. *Am. J. Physiol. Renal. Physiol.* 310 F621–F627. 10.1152/ajprenal.00344.2015 26764201 PMC4824142

